# Dynamic information system for small molecules

**DOI:** 10.1186/1758-2946-6-S1-P28

**Published:** 2014-03-11

**Authors:** Kiran K Telukunta, Xavier Lucas, Kersten Döring, Björn A Grüning, Stefan Günther

**Affiliations:** 1 Institute of Pharmaceutical Sciences, Research Group Pharmaceutical Bioinformatics, Freiburg, Baden-Württemberg, 79104, Germany

## 

The analysis of the biological effects of small molecules by mining lot of growing databases is an important task in the field of pharmaceutical sciences. To identify potential new similar drugs or to assess health risks from chemicals requires detailed knowledge about compounds.

The pursued project is aiming at the integration of existing data resources of small molecules that are combined with tools for the prediction of biological effects based on molecular interactions. Users can query the system for a given compound and retrieve information on questions such as:

• Which other similar compounds exist?

• Which proteins are mentioned in the literature in the same context of the given or similar compound(s)?

• Has the compound been tested in a bioassay?

• Is the compound toxic?

• Is the compound purchasable?

• Is the compound patented?

Answers to the above questions will be given by the web interface dynamically from the web server. The system further records the users’ choices and learns from them such that more important information shown more prominently.

The system is based on a large compound library consisting of above 70 million molecules collected from different publicly available resources (e.g., PubChem [1], ChEMBL [2], ZINC [3], StreptomeDB [4]) and will have the ability to accommodate new databases easily.

An integrated text mining tool (CIL [5]) provides information on functional relations of queried compounds to proteins and refers to articles describing them. Tools for the prediction of biological effects such as SuperPred [6] will also be included. Subsequently, the results will be adapted to the requirements of the user and gives an extensive digest of relevant information for pharmaceutical researchers.
